# Supplementary material for: Learning curve and surgical outcome of robotic assisted colorectal surgery with ERAS program
Source: Sci Rep. 2022 Nov 29;12:20566. doi: 10.1038/s41598-022-24665-w (PMC9709162; doi:10.1038/s41598-022-24665-w)
Supplement: Supplementary file 2 — Supplementary Information 2. [file 41598_2022_24665_MOESM2_ESM.zip › RAL Raw data-Ñ[▒K/133/Patho_ñw╝╨░O▒Kñσ.pdf]

臺中榮民總醫院 臺中市臺灣大道四段1650號 病理部活體組織切片 臨床醫學實驗及病理診斷中心 檢驗(查)報告

[redacted]

Pathologic diagnosis:
1. IMA root, dissection --- Unremarkable lymph nodes (0/3).
2. Sigmoid colon, robot-assisted colectomy --- Status post endoscopic mucosal resection with focal xanthomatous inflammation. No residual tumor is seen. The bilateral cut ends and mesocolic lymph nodes (0/12) are free of tumor.
3. Colon, cut end, distal, resection --- Unremarkable.

Prognostic and predictive factor:
(combined with previous specimen S1037605)
1. Tumor size: 0.3 cm.
2. Depth of invasion: Submucosa.
3. Histologic grade: G2: Moderately differentiated.
4. Angiolymphatic invasion: Not identified.
5. Perineural invasion: Not identified.
6. Tumor deposits (discontinuous extramural extension): Not identified.
7. Macroscopic tumor perforation: Not identified.
8. Surgical Margins:
a) Proximal Margin: Uninvolved by invasive carcinoma.
b) Distal Margin: Uninvolved by invasive carcinoma.
c) Radial or Mesenteric Margin: Uninvolved by invasive carcinoma, distance of tumor from margin cannot be assessed.
9. Lymph node status: Uninvolved (0/15).
10. Extranodal involvement: Not applicable.
11. Tumor regression grading S/P CCRT: No known presurgical therapy.
12. Type of polyp in which invasive carcinoma arose: Tubulovillous adenoma.
13. Additional pathologic findings: Not identified.
14. Pathological TNM Stage: pT1N0 (According to the eighth edition, American Joint Committee on Cancer Staging Guidelines for Tumors).
15. TNM descriptors: Not applicable.

Gross description:
The specimen consists of 1) IMA root, a piece of tan yellow soft tissue, 2.4x1.8x1.5 cm. 2) a segment of colon, 13.6 cm in length and 5.7 cm in proximal cut end circumference, 5.5 cm in distal circumference, and mesocolic soft tissue. No tumor, polypoid or ulcerative lesion is seen over the mucosa. There is a black stitch, 6.5 cm from the nearest margin. An area with black submucosal pigmentation, 2x1 cm, is seen 1 cm from the stitch. On cross section, no lesion is seen around the stitch site or pigmented area. The both cut ends are free of lesion grossly. 3) distal cut end, a piece of tan mucosa-covered soft tissue, 2.3x1.9x1 cm. Representative part for section: A) specimen 1 B) proximal cut end C) distal cut end D-E) stitch site F-G) pigmented area H) radial margin near stitch site J) specimen 3 K-L) lymph nodes of mesocolon.

Note:
This case has been peer reviewed by two doctors.
#T-59470_2 #M-09413_2 0 1105 SP0000
#T-C4560_2 #M-09183_2 0 1105 000000

已電子簽章

敬告：本報告僅供醫師參考使用，不具任何申請效力！！
請病患妥為保管，若不慎遺失需自負個人資料外洩責任！
